# Exosomal miR-10a derived from amniotic fluid stem cells preserves ovarian follicles after chemotherapy

**DOI:** 10.1038/srep23120

**Published:** 2016-03-16

**Authors:** Guan-Yu Xiao, Chun-Chun Cheng, Yih-Shien Chiang, Winston Teng-Kuei Cheng, I-Hsuan Liu, Shinn-Chih Wu

**Affiliations:** 1 Institute of Biotechnology, National Taiwan University, Taipei, Taiwan; 2 The Ph.D. Program for Cancer Biology and Drug Discovery, College of Medical Science and Technology, Taipei Medical University, Taipei, Taiwan; 3 Technology Commons, College of Life Science, National Taiwan University, Taipei, Taiwan; 4 Department of Animal Science and Technology, National Taiwan University, Taipei, Taiwan; 5 Department of Animal Science and Biotechnology, Tunghai University, Taichung, Taiwan; 6 Research Center for Developmental Biology and Regenerative Medicine, National Taiwan University, Taipei, Taiwan

## Abstract

Chemotherapy (CTx)-induced premature ovarian failure (POF) in woman remains clinically irreversible. Amniotic fluid stem cells (AFSCs) have shown the potential to treat CTx-induced POF; however, the underlying mechanism is unclear. Here we demonstrate that AFSC-derived exosomes recapitulate the anti-apoptotic effect of AFSCs on CTx-damaged granulosa cells (GCs), which are vital for the growth of ovarian follicles. AFSC-derived exosomes prevent ovarian follicular atresia in CTx-treated mice *via* the delivery of microRNAs in which both miR-146a and miR-10a are highly enriched and their potential target genes are critical to apoptosis. The down-regulation of these two miRNAs in AFSC-derived exosomes attenuates the anti-apoptotic effect on CTx-damaged GCs *in vitro.* Further, the administration of these miRNAs recapitulates the effects both *in vitro* and *in vivo*, in which miR-10a contributes a dominant influence. Our findings illustrate that miR-10a has potential as a novel therapeutic agent for the treatment of POF.

Chemotherapy (CTx) is designed to eliminate highly dividing cells by inducing DNA damage^1^ through the release of cytochrome c from the mitochondria to activate apoptotic pathways^2^. Consequently, CTx is commonly used for the treatment of various malignancies and can improve the survival rate of cancer patients^3^. Chemotherapeutic drugs may also damage other cells with physiologically high turnover rates, such as ovarian granulosa cells (GCs)^4^, which are required for oocyte survival and follicle development^5^. Hence, a potential side effect of CTx is a decrease in fertility that results in irreversible premature ovarian failure (POF)^6^,^7^,^8^, leading to hypergonadotropic hypogonadism and amenorrhoea in women under 40 years of age^9^,^10^,^11^,^12^. Furthermore, CTx has been proven to be associated with increased risks of cardiovascular disease^13^, osteoporosis^14^ and psychiatric diseases, such as depression^15^. To date, POF is clinically irreversible; therefore, advanced treatment strategies are urgently required.

Stem cell therapy has been suggested as a promising measure in the treatment of several human diseases and application of regenerative medicine because of their self-renewal and differentiation abilities to replace the damaged tissue or their capacities of producing paracrine factors to rescue injured tissues^16^. Recently, amniotic fluid stem cells (AFSCs) have been reported to have the ability to rescue ovarian function in a mouse model of CTx-induced POF^17^,^18^,^19^,^20^; however, the underlying mechanisms remain elusive. Our previous study implied that the therapeutic effects of AFSCs on CTx-damaged ovaries were likely derived from secretory factors, which support survival of ovarian follicles^17^. Because ovarian GCs are necessary to sustain follicle survival^21^,^22^,^23^ and are the major cell type that AFSCs encounter when injected into the ovary, it is probable that the interaction between AFSCs and GCs plays a key role in this favourable effect.

Exosomes are particles derived from the endosomal membrane partition after fusion with the plasma membrane; sizes range from 30 to 120 nm in diameter^24^. Recent studies proposed that exosome-mediated RNA delivery contributes to the regulation of the target cells fate^25^,^26^. Exosomes released from stem cells have been reported as a novel means of a specific cell-to-cell interaction that has a beneficial effect on stem cell therapy^27^. Accordingly, the identification of exosome-mediated interaction between stem cells and target cells may provide novel strategies for treating POF in the clinic.

In this study, we revealed that AFSC-derived exosomes contained two microRNAs (miRNAs), miR-146a and miR-10a, which inhibited apoptosis in damaged GCs and prevented ovarian follicles from atresia in mice following CTx. We found that the knockdown of miR-146a and miR-10a expression in AFSCs attenuated the therapeutic effects of AFSC-derived exosomes on damaged GCs *in vitro*. We also showed that direct delivery of miR-10a in CTx-induced mice *via* liposomes effectively repressed apoptosis in ovarian cells and rescued follicles from atresia. These findings shed new light on the role of miR-10a in the restorative process and imply the promise of a cell-free therapeutic strategy for POF treatment.

## Results

### Therapeutic effects of AFSCs secreted factors

Chemotherapeutic drugs induce ovarian damage mainly through the cytotoxicity of its metabolite to GCs *in vivo*, called phosphoramide mustard (PM)^28^, which is highly unstable. Here we used nitrogen mustard (NM) which is structurally and functionally similar to PM^29^ to induce the damage of GCs in experiments *in vitro*. To determine the effects of AFSCs secreted factors on NM-damaged GCs, we co-cultured these cells with AFSCs using a co-culture transwell system. After 48 hours of co-culture, apoptosis in NM-induced GCs was significantly reduced when co-cultured with AFSCs, but these results were not found when cells were co-cultured with NIH-3T3, which are mouse fibroblast cells (Supplementary Fig. 1). To confirm whether the effect was derived from the secretory factors of AFSCs, we also cultured GCs directly with the conditioned medium (CM) from AFSCs after the NM treatment. Our results show that NM-damaged GC viability was sustained when co-cultured with AFSCs and AFSC-derived CM (Supplementary Fig. 2). In addition, AFSCs and AFSC-derived CM significantly suppresses apoptosis in damaged GCs (Supplementary Fig. 3). These findings indicate that the therapeutic effect of AFSCs predominantly results from the release of secretory factors.

### Contribution of AFSC-derived exosomes to anti-apoptosis in damaged GCs

To assess if exosomes play a restorative role *via* transferring of RNAs, we isolated exosomes from AFSC-derived CM and treated them with or without RNase. In our study, AFSC-derived exosomes were confirmed by electron microscopy and Nano C Analyzer as round structures of approximately 30–120 nm (Supplementary Fig. 4a,b) and were labelled with PKH26, a lipophilic fluorescent dye, to track exosomes in the following studies (Supplementary Fig. 4c). Bioanalyzer analysis showed that the RNA content of exosomes diminished following RNase treatment (Supplementary Fig. 4d). After 3 hours of culture with AFSC-derived exosomes, both RNase-treated and -untreated exosomes (30 μg ml^−1^ of exosomes proteins) were incorporated in damaged GCs and ubiquitously distributed throughout the cytoplasm (Supplementary Fig. 4e). We further demonstrated that with a high enough quantity of exosomes proteins (>5 μg ml^−1^) intact exosomes could significantly sustain survival and reduce apoptotic cell death in damaged GCs compared with the CTx-only group (Supplementary Fig. 4f,g). In contrast, RNase treatment abolished this protective effect (Supplementary Fig. 4f,g). These results demonstrate that the transfer of RNAs *via* AFSC-derived exosomes to damaged GCs plays an important role in therapy.

### AFSC-derived exosomes rescues GCs from apoptosis and preserves ovarian follicles in CTx-treated mice

To further address whether the effects of exosomes are similar to AFSCs in the ovaries from CTx-treated mice^17^, we directly injected either AFSCs or AFSC-derived exosomes (with or without RNase treatment) into ovaries of our mouse models (Supplementary Fig. 5a). At day 1 (24 hours after CTx), discernibly more follicular cells underwent apoptosis until day 8 when the apoptotic level of ovarian GCs dropped to basal levels in CTx-phosphate-buffered saline (PBS)-mice (Supplementary Fig. 5b). In CTx-AFSC-mice at day 2 (after AFSC transplantation for 24 hours), we found that the apoptotic level was significantly repressed compared with that in CTx-PBS-mice; the effect lasted until day 8. Meanwhile in CTx-exosomes (Exo)-mice, the results were similar to the effect of AFSCs until day 3 (Supplementary Fig. 5b). Nevertheless, exosomes with RNase treatment (ExoR) completely abolished this anti-apoptotic effect (Supplementary Fig. 5b).

During this experiment, the number of primordial follicles was significantly higher in CTx-AFSC-mice and CTx-Exo-mice at days 3 and 8 than that in CTx-PBS-mice. However, there was no difference between CTx-PBS-mice and CTx-ExoR-mice at all time points (Supplementary Fig. 5c). CTx-AFSC-mice preserved more total healthy follicles than CTx-PBS-mice did at days 3 and 8, whereas there were indistinguishable effects in CTx-Exo-mice (Supplementary Fig. 5d). Furthermore, AFSCs significantly reduced the numbers of atretic follicles at day 2 up to 8, while injection of the exosomes recapitulated this result (Supplementary Fig. 5e). In contrast, RNase treatment disrupted this beneficial outcome (Supplementary Fig. 5e). These results indicate that AFSC-derived exosomes could prevent ovarian follicular atresia and sustain follicular development in CTx-mice *via* the delivery of RNAs.

### Therapeutic effects on damaged GCs by exosomal microRNAs derived from AFSCs

**Figure 1:**
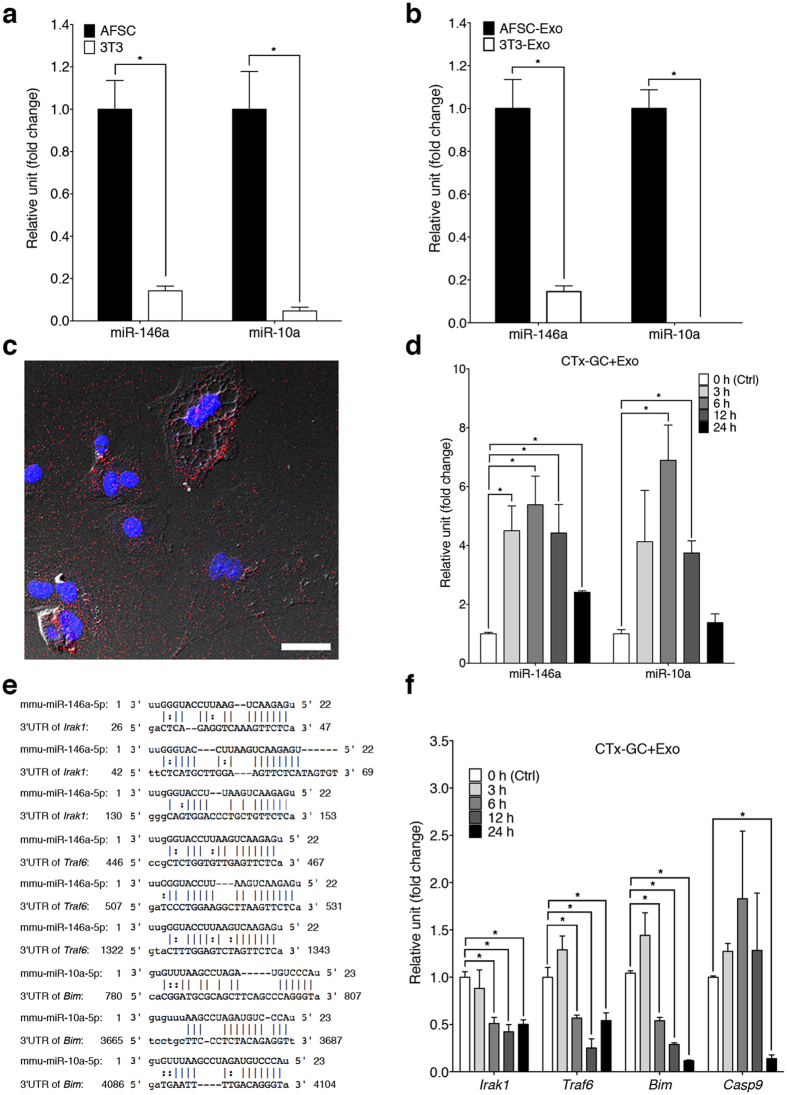
Delivery of miRNAs into damaged GCs via exosomes. (**a**,**b**) qRT-PCR analysis of expression levels of miR-146a and miR-10a in AFSCs and NIH-3T3 (**a**) and their corresponding exosomes (**b**). Error bars represent s.e.m. *n* = 3. *P < 0.05; unpaired *t* test. (**c**) A representative micrograph shows that PKH26 labeled-AFSC-derived exosomes (30 μg ml^−1^ of exosomes proteins) (red) were incorporated into the cytoplasm of damaged GCs. Nucleus was stained by DAPI (blue). Scale bar, 40 μm. (**d**) The levels of miR-146a and miR-10a in damaged GCs cultured with AFSC-derived exosomes at different time points compared to 0 h (Ctrl). Error bars represent s.e.m. *n* = 3. *P < 0.05; unpaired *t* test. (**e**) The sequence alignment of miR-146a and its predicted target sites of the mouse *Irak1* and *Traf6* mRNA 3′**-**untranslated region (3′**-**UTR), and miR-10a and its putative target sites of the mouse *Bim* mRNA 3′**-**UTR. (**f**) The expression levels of *Irak1*, *Traf6, Bim and Casp9* in damaged GCs cultured with AFSC-derived exosomes at different time points compared to 0 h (Ctrl). Error bars represent s.e.m. *n* = 3. *P < 0.05; unpaired *t* test.

To identify candidate RNAs involved in the AFSC-dependent therapeutic effect, we analysed the composition of RNAs in AFSC-derived exosomes and compared them with the expression profiles of the exosomes derived from NIH-3T3, which showed no substantial benefits to damaged GCs (Supplementary Fig. 1). We found 114 miRNAs that were increased in AFSC-derived exosomes (Supplementary Table 1). To verify the accuracy of the results, we selected miR-146a and miR-10a, the most highly enriched miRNAs in AFSC-derived exosomes (287.20- and 54.96-fold respectively; Supplementary Table 1), for further validation by quantitative reverse transcription (qRT)-PCR. The expression of both miR-146a and miR-10a was significantly increased in AFSCs and in AFSC-derived exosomes than that in NIH-3T3 and NIH-3T3-derived exosomes (Fig. 1a,b). Hence, we used miR-146a and miR-10a for the further investigation, since they both are associated with the suppression of cell apoptosis^30^,^31^.

**Figure 2:**
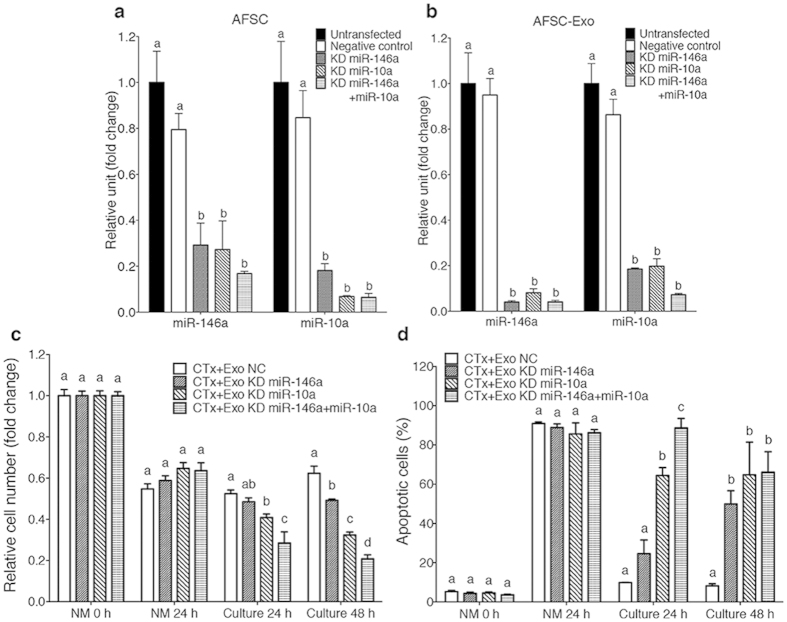
Down-regulation of miR-146a and/or miR-10a impaired the effects of AFSC-derived exosomes on damaged GCs *in vitro*. (**a**,**b**) qRT-PCR analysis showed that the expression of miR-146a and miR-10a was knocked down in AFSCs (**a**) and AFSC-derived exosomes (**b**) when AFSCs were transfected with miR-146a or/and miR-10a inhibitors. Error bars represent s.e.m. *n* = 3. Different characters (a,b) represent significant differences (*P* < 0.05) among each group; Tukey’s multiple comparisons test. (**c**) The fold change of relative cell number of damaged GCs cultured with AFSC-derived exosomes with various transfections at different time points. Error bars represent s.e.m. *n* = 6. Different characters (a–c) represent significant differences (*P* < 0.05) among each group; Tukey’s multiple comparisons test. (**d**) The percentage of apoptotic cells of damaged GCs cultured with AFSC-derived exosomes with various transfections at different time points. Error bars represent s.e.m. *n* = 3. Different characters (a–c) represent significant differences (*P* < 0.05) among each group; Tukey’s multiple comparisons test.

To validate that AFSCs could deliver miR-146a and miR-10a to damaged GCs *via* exosomes, we cultured damaged GCs with AFSC-derived exosomes. After 3 hours of culture, labelled exosomes (30 μg ml^−1^ of exosomes proteins) were incorporated in damaged GCs (Fig. 1c) and the levels of the miRNAs in GCs subsequently increased (Fig. 1d). We then predicted the targets of miRNAs using miRBase/miRanda, and it revealed that *Irak1* and *Traf6* are potential target genes of miR146a while *Bim* is the putative target gene of miR-10a (Fig. 1e). Interestingly, IRAK1, TRAF6 and BIM have been reported to be involved in apoptotic pathways^32^,^33^. Meanwhile, qRT-PCR demonstrated that the expression levels of these genes and *caspase 9* (*Casp9*)^33^, the downstream gene of BIM, in damaged GCs decreased dramatically after culture with AFSC-derived exosomes (Fig. 1f). We next examined whether miR-146a and miR-10a contributed therapeutic value. First, we knocked down the expression of miR-146a or/and miR-10a in AFSCs or in AFSC-derived exosomes using miRNA inhibitors and confirmed expression by qRT-PCR (Fig. 2a,b). Interestingly, the knockdown of miR-146a resulted in the down-regulation of miR-10a and *vice versa* (Fig. 2a,b). We then incubated damaged GCs with the exosomes, which could be internalised by damaged GCs in 3 hours (Supplementary Fig. 6). Our results show that the down-regulation of miR-146a or miR-10a attenuated the efficacy of sustaining survival in damaged GCs compared with that in negative control, whereas the double knockdown of both miRNAs impaired the effect more prominently (Fig. 2c). Between the two miRNAs, the knockdown of miR-146a in exosomes ablated the anti-apoptotic properties in damaged GCs after 48 hours of culture compared with that in the negative control, whereas the down-regulation of miR-10a disrupted this activity at as early as 24 hours (Fig. 2d). Moreover, the double knockdown of both miRNAs reduced the effect more significantly after 24 hours of culture; conversely, there was no difference between double and single down-regulation after 48 hours of culture (Fig. 2d). These results suggest that miR-146a and miR-10a could have a critical role in the restorative effect of AFSCs.

### Liposome-mediated delivery of microRNAs exerts anti-apoptotic functions in damaged GCs

**Figure 3:**
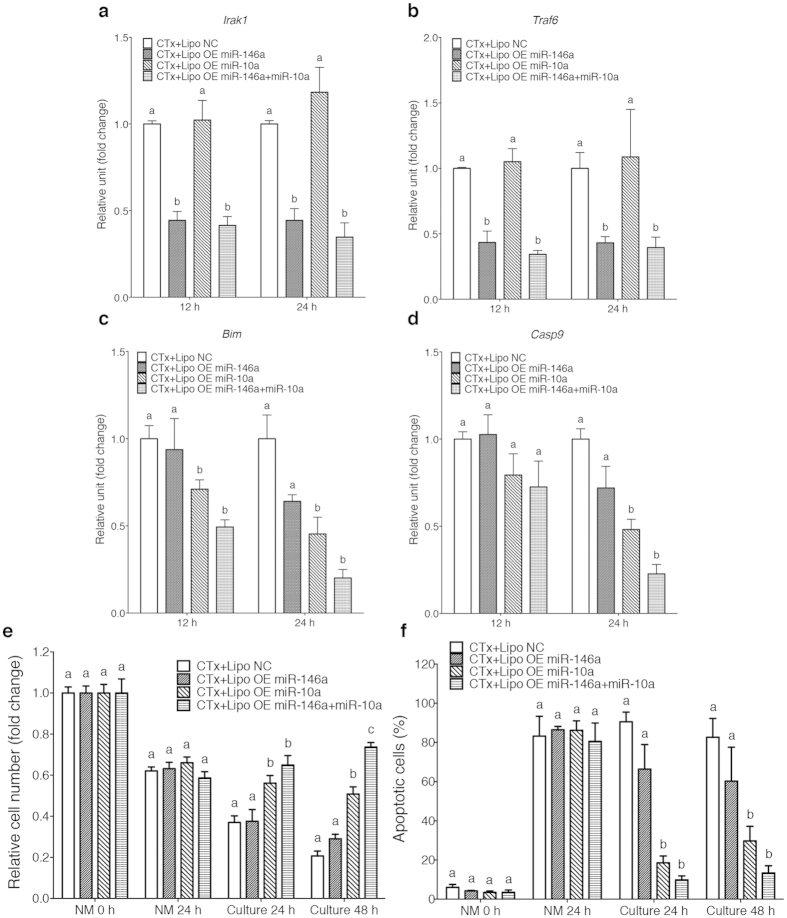
MiR-10a recapitulates the effects of AFSC-derived exosomes on damaged GCs *in vitro*. (**a–d**) The expression levels of *Irak1* (**a**), *Traf6* (**b**), *Bim* (**c**) and *Casp9* (**d**) of damaged GCs cultured with liposomes with various cargos at different time points. Error bars represent s.e.m. *n* = 3. Different characters (a–c) represent significant differences (*P* < 0.05) among each group; Tukey’s multiple comparisons test. (**e**) The relative cell number (fold change) of damaged GCs cultured with liposomes with various cargos at different time points. Error bars represent s.e.m. *n* = 6. Different characters (a–c) represent significant differences (*P* < 0.05) among each group; Tukey’s multiple comparisons test. (**f**) The percentage of apoptotic cells of damaged GCs cultured with liposomes with various cargos at different time points. Error bars represent s.e.m. *n* = 3. Different characters (a–c) represent significant differences (*P* < 0.05) among each group; Tukey’s multiple comparisons test.

Next, we further examined whether miR-146a or miR-10a itself can similarly reproduce the actions of AFSC-derived exosomes in damaged GCs. We directly delivered miR-146a or/and miR-10a to damaged GCs *via* artificial liposomes, which are lipid vesicles with similar characteristics to exosomes, for nucleic acid delivery^34^. Resembling AFSC-derived exosomes, PKH26-labelled liposomes with different cargos were endocytosed by damaged GCs after 3 hours of incubation (Supplementary Fig. 7). The expression levels of miR-146a target genes, *Irak1* and *Traf6*, in damaged GCs were significantly decreased 12 hours after miR-146a delivery and this effect was sustained for 24 hours (Fig. 3a,b). A similar result was also observed in the expression level of *Bim*, the predicted miR-10a target gene, 12 hours after miR-10a delivery (Fig. 3c). The downstream gene of *Bim*, *Casp9*, was significantly reduced 24 hours after miR-10a delivery (Fig. 3d). Furthermore, we found that miR-10a delivery sustained the number of GCs and reduced the number of apoptotic GCs following NM treatment after 24 and 48 hours of culture, whereas delivering both miR-146a and miR-10a only improved the viability of GCs after 48 hours of culture but did not improve the reduction of apoptosis in GCs when compared with that in miR-10a-only group (Fig. 3e,f).

### Liposome-mediated miRNA delivery prevents follicle atresia in CTx-treated mice

**Figure 4:**
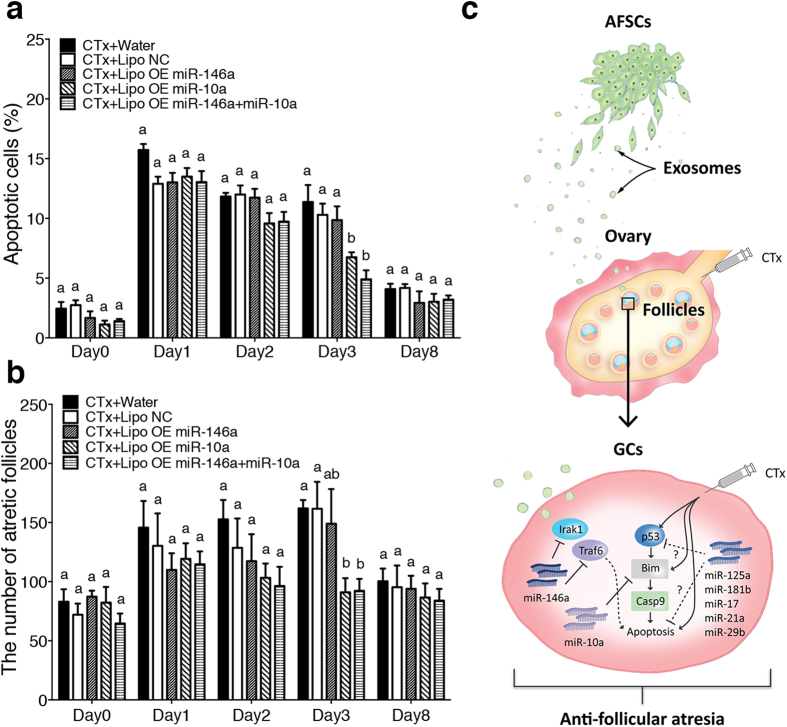
MiR-10a exerts beneficial effects on ovaries of CTx-mice. (**a**) The percentage of apoptotic cells in the ovaries of each group at different time points. Error bars represent s.e.m. *n* = 3. Different characters (a,b) represent significant differences (*P* < 0.05) among each group; Tukey’s multiple comparisons test. (**b**) Atretic follicles were counted in the ovarian sections of each group at different time points. Error bars represent s.e.m. *n* = 3. Different characters (a,b) represent significant differences (*P* < 0.05) among each group; Tukey’s multiple comparisons test. (**c**) A schematic diagram of the restorative mechanism of AFSCs on damaged GCs. AFSCs can restore the fertility and prevent POF in CTx treated mice possibly by delivering miR-146a, miR-10a and other potential miRNAs via exosomes to damaged GCs, and in turn down-regulating the pro-apoptotic genes. The dotted line represents the putative interactions between Irak1/Traf6 and the apoptotic pathway and that the potential miRNAs contribute to anti-apoptosis.

To further assess whether miRNAs have the capacity to preserve follicles in CTx-treated mice, we directly injected PKH26-labelled liposomes carrying miR-146a or/and miR-10a into bilateral ovaries of CTx-treated mice. The ovarian cells internalised the liposomes 6 hours after injection, and the labelled signals persisted strongly for at least 48 hours and disappeared after 7 days (Supplementary Fig. 8). At day 3, 48 hours after the injection, the delivery of miR-10a or both miR-146a and miR-10a miRNAs significantly repressed the apoptosis, and there was no significant difference between these two groups (Fig. 4a). Similarly, delivery of only miR-10a or both miR-146a and miR-10a miRNAs significantly suppresses follicular atresia at day 3, whereas there was no significant difference among each group at other time points (Fig. 4a). These findings indicate that miR-10a plays a pivotal role in the therapeutic effects of AFSCs in CTx-induced ovarian failure.

## Discussion

The amenorrhea incidence rate of CTx in young women by the end of treatment is 84% and eventually half of patients go on to develop POF^35^. To date, there is no effective measure to prevent POF; therefore, there is an urgent need for new treatment strategy. Acute follicle atresia is induced by CTx *via* extensive apoptotic cell death in GCs^4^, predominantly within 72 hours after treatment^36^. Consequently, the ensuing increase in the recruitment of primordial follicles and, in turn, the decline in the number of all types of follicles ultimately results in POF^37^. Our previous study demonstrated that the transplantation of AFSCs preserved more ovarian primordial follicles in CTx-induced POF mice thus extending the reproductive life in CTx-mice^17^. Here, we first show that the benefits of AFSCs to the damaged GCs were mainly derived from the secretory factors. Subsequently, our data illustrated that exosomes derived from AFSCs were able to support survival and to elevate the apoptotic resistance of damaged GCs *in vitro*. However, the effective duration for exosomes to inhibit ovarian cells apoptosis *in vivo* was shorter than that for AFSCs. We suppose that this result was because of the short half-life of exosomes *in vivo*^38^. Despite the reduced duration of efficacy, AFSC-derived exosomes exerted anti-apoptotic activity in ovarian cells within 72 hours after CTx; as a result, the exosomes helped preserve more ovarian follicles. The pretreatment of the exosomes with RNase disrupted the *in vitro* and *in vivo* advantages of the exosomes, indicating that RNAs transferred by exosomes contributes to the therapeutic effects. These results are consistent with other studies reporting that the delivery of RNAs *via* exosomes is a novel mechanism that is critical in various beneficial properties of stem cell-based therapy^27^,^39^.

A key issue we addressed in the present study is the identification of miRNAs in AFSC-derived exosomes. Chemotherapeutic agents have been reported to stimulate p53 activation and its downstream targets, such as Bim and Casp9, that result in apoptosis^40^,^41^. It has been proven that miR-146a relates to the regulation of cell apoptosis *via* the down-regulation of the expression of its targets, Irak1 and Traf6^32^. In addition, miR-10a has been shown to modulate cell apoptosis through the suppression of pro-apoptotic factor, Bim^31^. Our data demonstrated the increased levels of miR-146a and miR-10a as well as the decreased levels of the target genes of these miRNAs in CTx-damaged GCs after culture with AFSC-derived exosomes. These results indicate that the two exosomal miRNAs play crucial roles in the anti-apoptotic effects on CTx-damaged GCs.

To further confirm the function of the exosomal miRNAs, we performed knockdown studies in AFSC-derived exosomes. Accordingly, after down-regulating the expression of the miRNAs in AFSCs, we predicted that the miRNAs expression of their respective exosomes would also be reduced. The results met our expectation, expression levels of miR-146a and miR-10a were suppressed in both of the producer cells and their exosomes when RNA inhibitors were administered. Interestingly, we observed a potential link between miR-146a and miR-10a. The down-regulation of miR-146a led to a reduction in miR-10a expression and *vice versa,* which is in accordance with a previous study^42^. Several groups have reported that miR-146a functions in repressing NF-κB pathway^43^,^44^, which has been shown to negatively regulate the expression of miR-10a^45^. Moreover, recent studies have evidenced that miR-10a targets Bcl-6^46^, which results in down regulation of miR-146a^47^. It is possible that the observation in our study may be attributed to the regulation between NF-κB pathway and Bcl-6. However, further studies are needed to clarify whether the interaction is mediated directly by NF-κB pathway and Bcl-6 or indirectly through interacting with other pathways.

According to previous studies, miR-146a can potentially rescue damaged cells in various injury models^30^,^48^, and miR-10a is associated with the regulation of cell apoptosis in human cumulus-oocytes complex^49^. In our study, knocking down miR-146a and miR-10a reduces the therapeutic effects of AFSC-derived exosomes implying these two miRNAs have essential roles in exosome activity in damaged GCs. Nevertheless, the delivery of miR-146a reduced the average level of GC apoptosis comparing to negative control (NC) (from around 90% to 66% at 24 hours of culture; from around 82% to 60% at 48 hours of culture) with no statistical difference (Fig. 3f) and that the delivery of both miRNAs only slightly reduced the average level of GC apoptosis comparing to miR-10a alone (from around 18% to 9% at 24 hours of culture; from around 29% to 13% at 48 hours of culture) with no significant difference between the combination and miR-10a alone (Fig. 3f). In contrast, when miR-10a was directly delivered to damaged GCs *in vitro* or to ovaries in CTx-mice, it exerted the effectively anti-apoptotic effect as its target genes silenced. According to previous reports and our data, miR-10a directly targets *Bim* and results in the down regulation of *Casp9*, which are crucial factors in apoptotic pathway^33^. Although there is evidence that the target of miR-146a, *Irak1* and *Traf6* are associated to apoptotic pathway^32^, the elaborate interaction between these genes and apoptosis is still elusive. Thus, we propose that miR-146a may play a minor role in the therapeutic effects through an indirect way, while miR-10a acts as a predominant factor.

Additionally, we noticed that the therapeutic effects of the delivery of miR-146a and miR-10a via liposomes were lower than that of exosomes *in vivo*. One possible explanation to this outcome could be due to the potentially lower delivery efficiency of liposomes compared to the exosomes. Alternatively, it is also possible that additional factors carried by exosomes might contribute to the beneficial effects as well. According to recent studies, miR-125a involves in the repression of cell apoptosis via directly targeting p53^50^ and that miR-181b reduces apoptosis^51^. Moreover, other miRNAs such as miR-17, miR-21a and miR-29b also contribute to anti-apoptosis through targeting various genes involved in apoptotic pathway^52^. Interestingly, these miRNAs also highly enriched in AFSC-derived exosomes (Supplementary Table 1), and it is possible that transferring not only miR-10a and miR-146a but also these miRNAs via AFSC-derived exosomes to damaged GCs rescues the cells from apoptosis through suppressing the apoptotic-related target genes and then prevents follicles from atresia (Fig. 4c).

Over the past few years, several studies have highlighted the potential therapeutics of using regulatory RNAs as a cure for various diseases or injury^47^,^49^,^50^,^51^. Our findings here also support this possibility. Nevertheless, with respect to clinical treatment, several concerns remained to be addressed. First, miRNAs play complicated roles in a variety of physiological processes in the body for maintenance of homeostasis^53^, and systematic administration of high dose of miRNAs may cause serious side effects. Local injection of these miRNAs into sites of injury and their short half-life may be beneficial to overcome this challenge. Second, the liposome-based delivery has been criticised for is toxicity to cells, non-specific targeting and potential immunogenicity^54^; therefore, optimisation of the system is needed to reduce risk and improve efficiency. Development of a novel delivery system may be a suitable alternative. In addition, it would be important to determine the appropriate timing for treatment. According to our data, the protection of GCs against apoptosis within the crucial 72 hours after CTx is a key period for the prevention of POF. It may be favourable to administer the miRNAs closer to or even prior to CTx so that maximal efficacy can be achieved.

Taken together, from the standpoint of translational medicine, our data provide the possibility of a novel clinical cell-free therapeutic as opposed to the administration of stem cells in patients, which avoids the concerns of unstable cell sources and the safety of allogenic donor cells. Our findings imply that the delivery of miR-10a could be useful for the preservation of ovarian follicles in female patients after CTx.

## Methods

### Mice

ICR mice aged 4–6 weeks were purchased from the Laboratory Animal Center of Medical College of National Taiwan University (Taipei, Taiwan). EGFP transgenic mice that ubiquitously express EGFP were previously described^55^. In brief, EGFP-expressing transgenic mouse lines were created by pronuclear microinjection of ICR strain zygotes with a construct (pCX-EGFP) that expresses EGFP under control of the *β-actin* promoter^56^,^57^,^58^. Mice were housed under a 14 to 10 h light-dark cycle at 25 °C ± 2 °C with food and water provided ad libitum. All experimental procedures with mice were approved by the Institutional Animal Care and Use Committee of National Taiwan University (NTU-102-EL-10). The methods were carried out in accordance with the approved guidelines.

### Isolation and culture of AFSCs

Mice were euthanized by sequential CO_2_ asphyxiation and cervical dislocation at day 11.5 of pregnancy. The uterus was isolated and freed of soft tissues, and 26-gauge needles were used to collect amniotic fluid from each EGFP-expressing or wild-type conceptus separately. Amniotic fluid was added to the wells of a 24-well cell culture dish (#92024, TPP, Trasadingen, Switzerland) containing 1 ml of Minimum Essential Medium alpha (MEM-α; #M0894, Sigma-Aldrich, St. Louis, MO) supplemented with 3.7 mg ml^−1^ NaHCO_3_ (#S5761, Sigma-Aldrich), 10% (v/v) fetal bovine serum (FBS) (#SH30070-03, Thermo Fisher Scientific, Waltham, MA), 100 IU ml^−1^ penicillin and 100 mg ml^−1^ streptomycin (#15140, Thermo Fisher Scientific). Cells were incubated at 37 °C in a humidified atmosphere of 5% CO_2_. Every three days, 0.5 ml of nonadherent cells were removed and replaced with 0.5 ml of fresh medium. Cells were passaged after reaching 70% to 80% confluence after incubation at 37 °C with 0.25% (w/v) trypsin/ethylenediaminetetraacetic acid (EDTA) (#2500056, Thermo Fisher Scientific) for 5 min. Flow cytometric analysis showed that cultured AFSCs expressed CD29, CD44, Sca-1, and MHC-I but did not express MHC-II, hematopoietic lineage markers (CD11b, CD34 and CD45) or an endothelial cell marker (CD31). Moreover, AFSCs displayed mesoderm trilineage differentiation capacity^17^. All the experiments were performed with AFSCs at passages three to five.

### Isolation and culture of GCs

Gonadotrophin from pregnant mare serum (PMSG, 10 IU) (#G4877, Sigma-Aldrich) was injected intraperitoneally into 6-week-old ICR female mice. After 44 to 48 h, the animals were euthanized and the ovaries were collected and 30-gauge needles were used to puncture the pre-ovulatory follicles and obtain GCs under stereomicroscope. GCs were culture in 6-well cell culture dishes (#92006, TPP) containing 2 ml of MEM-α supplemented with 3.7 mg ml^−1^ NaHCO_3_, 10% (v/v) FBS, 100 IU ml^−1^ penicillin and 100 mg ml^−1^ streptomycin. Cells were incubated at 37 °C in a humidified atmosphere of 5% CO_2_. Every three days, 2 ml of non-adherent cells were removed and replaced with 2 ml of fresh medium. All the experiments were performed with GCs at the first to third passages.

### Induction of GCs apoptosis *in vitro* and co-culture GCs with AFSCs

Nitrogen mustard (NM) (#MT-090916, Matrix Scientific, Columbia, SC) was dissolved in the GCs culture medium at 3 μM to induce apoptosis. Twenty-four hours after the exposure to NM, the GCs were co-cultured with AFSCs at a 1:5 ration of AFSCs to GCs in the transwell system (#PIEP30R48, Merck Millipore, Darmstadt, Germany) in which the GCs and AFSCs shared the same medium but kept separately, with the GCs seeded below the AFSC.

### TUNEL assay

GCs were fixed with 4% (w/v) paraformaldehyde (PFA) (#P6148, Sigma-Aldrich) for 15 min at room temperature and permeabilized using 0.25% Triton X-100 (v/v) (#0694, Amresco, Solon, OH) for 10 min. Apoptotic cells were evaluated by *in situ* Cell Death Detection Kit, TMR red (#12156792910, Roche, Mannheim, Germany) according to the manufacturers’ instructions. Nuclei were stained with 0.1 mg ml^−1^ 4′,6-diamidino-2-phenylindole (DAPI) (1:1000, #40043, Biotium, Hayward, CA) for 3 min. Cells were observed using a fluorescence microscope (DMIRB; Leica, Wetzlar, Germany). GCs were counted with ImageJ software (version 1.48, NIH) in three randomly selected fields in every well of the cell culture dish to quantify the apoptotic level, defined as the percentage of apoptotic GCs.

For histological assay, ovaries were fixed with 4% PFA at 4 °C overnight, incubated in 30% sucrose (#S0389, Sigma-Aldrich) at 4 °C for three days, frozen, serially sectioned (8 μm), and incubated with PBS containing 0.25% Triton X-100 for 15 min. Apoptotic cells were assessed by *in situ* Cell Death Detection Kit, TMR red according to the manufacturers’ instructions. Nuclei were stained with DAPI for 3 min. All sections were mounted with fluoroshield mounting medium (#ab104135, Abcam, Cambridge, UK) on glass slides and observed using a fluorescence microscope. GCs were counted with ImageJ software in three randomly selected fields in every tenth section with random start to estimate the apoptotic level, defined as the percentage of apoptotic GCs.

### Cell viability assay

Add 3-(4, 5-dimethylithiazol-2-yl)-2, 5-diphenyl tetrazolium bromide (MTT) (#M2128, Sigma-Aldrich) (5 mg ml^−1^) into culture medium and incubate cells for 4 h at 37 °C. Remove medium and then add dimethyl sulfoxide (DMSO) (#D2650, Sigma-Aldrich) to incubate cells for 6 min at RT. Measure the absorbance at 570 nm and 690 nm by SpectraMax 190 ELISA plate reader (Molecular Devices, Sunnyvale, CA).

### Isolation of AFSC-derived exosomes

Exosomes were obtained from conditioned medium (CM) of AFSCs by using the ExoQuick-TC (#EXOTC50A-1, SBI, Mountain View, CA). In brief, AFSCs at approximately 80% confluence were cultured for 48 h and the CM were collected to centrifuge at 2000 *g* for 20 min and filtrated through 0.22 μm filters to remove cell debris. Ten ml of supernatant was mixed 2 ml of ExoQuick precipitation solution and incubated overnight at 4 °C. After incubation, exosomes were centrifuged at 5000 *g* for 30 min to form pellet. The exosomes pellet was resuspended in 100 μl phosphate buffered saline (PBS) (#0780, Amresco). In selected experiments, exosomes were treated with RNase A (100 μg ml^−1^, #AXP025, ARROW TEC, Rolla, ND) for 1 h at 37 °C, the reaction was stopped by the addition of the RNase inhibitor (400 U ml^−1^, #10777-019, Invitrogen, Carlsbad, CA) and exosomes were washed by centrifugation. The effectiveness of RNase treatment was examined by total RNA extraction using the TRIzol reagent (#10296-010, Invitrogen) and subjected to Bioanalyzer analysis (Agilent 2100 bioanalyzer, Agilent Technologies, Santa Clara, CA) with the Agilent total RNA PicoChip to confirm the degradation of RNA after RNase treatment according to the manufacturers’ instructions. The exosomes were labeled with PKH26 (#MINI26, Sigma-Aldrich), a fluorescent dye intercalating into lipid region, according to the manufacturers’ instructions. After labeling, the exosomes pellet was resuspended in 100 μl PBS and the protein content was quantified by the BCA method (#23225, Thermo Fisher Scientific) according to the manufacturers’ instructions. The AFSC-derived exosomes without or with RNase treatment were directly added into culture medium of CTx-damaged GCs at various concentrations of exosomes proteins.

### Electron microscopy analysis of AFSC-derived exosomes

The exosomes pellet derived from AFSCs was dissolved in PBS, loaded to copper grids, stained with 1% (w/v) phosphotungstic acid (PTA) (#19500, Electron Microscopy Sciences, Washington, PA), and then examined by transmission electron microscopy with an accelerating voltage of 75 kV (H-7650, HITACH, Tokyo, Japan).

### Delsa Nano C Analyzer analysis

The size distribution of AFSC-derived exosomes was determined using Delsa Nano C Analyzer (DelsaNano C, Beckman Coulter, Brea, CA) according to the manufacturers’ instructions.

### Mouse model of CTx-induced ovarian failure

Six-week-old ICR female mice were administered busulfan (20 mg kg^−1^) (#B2635, Sigma-Aldrich) and cyclophosphamide (200 mg kg^−1^) (#C0768, Sigma-Aldrich) dissolved in DMSO using a single intraperitoneal injection or only injection with DMSO as the vehicle control.

### AFSCs transplantation and exosomes injection

After CTx for 24 h, PBS only, EGFP-AFSCs or AFSC-derived exosomes without or with RNase treatment were transplanted into the ovaries of CTx-mice according to the previous study^17^. In brief, a suspension of 5 × 10^5^ EGFP-AFSCs or 125 μg of exosomes proteins (an approximate amount produced by 5 × 10^5^cells overnight) in 5 μl of sterilized PBS were injected through a glass pipette (100 μm tip) into each ovary of mice anesthetized with inhaled isoflurane (#B506, Abbott, Chicago, IL). CTx-mice with the injection of PBS, EGFP-AFSCs or AFSC-derived exosomes without or with RNase treatment were designated to CTx-PBS-, CTx-AFSC-, CTx-Exo- and CTx-ExoR-mice.

### Ovarian morphologic and follicle counts

In brief, ovaries were fixed with 4% PFA at 4 °C overnight, embedded in paraffin, serially sectioned (8 μm), and stained with hematoxylin-eosin. The number of primordial, total healthy, and atretic follicles in every tenth section with random start were recorded. The different categories of follicles were determined as described previously^59^. Only follicles containing an oocyte with a clearly visible nucleus were scored.

### Next generation sequencing (NGS) analysis

Total RNA of exosomes derived from AFSCs or NIH-3T3 was extracted using the TRIzol reagent and conducted to process Bioanalyzer analysis using the Agilent total RNA PicoChip to determine the RNA quantity and quality according to the manufacturers’ instructions. Small RNA libraries were constructed using the TruSeq Small RNA Sample Preparation Kit (#RS-200-0012, Illumina, San Diego, CA), and sequencing was performed for each sample on the MiSeq Desktop Sequencer (Illumina) according to the manufacturers’ instructions. Raw reads were first extracted from FASTQ files and trimmed adapter using skewer. The prepared reads were filtered and sequences with lengths from 15 to 55 bp were aligned using CLC Genomics Workbench (version 7.5.1, CLC bio, Aarhus, Denmark) against the mouse miRNA sequences downloaded from miRBase (Release 21). The miRNA expression level was normalized as reads per kilo base per million mapped reads (RPKM). The altered transcripts were compared using CLC Genomics Workbench.

### Reverse transcription and quantitative reverse transcription polymerase chain reaction

The expression level of mature miR-146a and miR-10a in AFSCs, NIH-3T3 and their corresponding exosomes was analyzed by qRT-PCR. After total RNA extraction, all samples were reverse transcribed into cDNA using the miScript II RT Kit (#218161, Qiagen, Venlo, Netherland) and qRT-PCR was performed using the miScript SYBR Green PCR Kit (#218073, Qiagen) according to the manufacturer’s instructions. The miScript Primer Assays were used to assess the expression level of miR-146a and miR-10a (#MS00001638 for miR-146a and #MS00032242 for miR-10a, Qiagen), whereas RNU6-2 (#MS00033740, Qiagen) was used as an endogenous control. The expression level of miR-146a, miR-10a and their predicted target mRNAs in GCs cultured with 30 μg ml^−1^ of exosomes proteins were analyzed by qRT-PCR according to the manufacturer’s instructions. The sequences of primers for assessing the levels of target mRNAs were shown in Supplementary Table 2. The expression level of microRNAs and mRNAs was measured by qRT-PCR using a CFX96 Touch™ Real-Time PCR Detection System (Bio-Rad, Hercules, CA).

### Inhibitor-mediated knockdown of miRNA expression in AFSCs

AFSCs were used for inhibitor transfection, targeting miR-146a, miR-10a or both. Inhibitors (#MIN0000158 for miR-146a and #MIN0000648 for miR-10a, Qiagen) and inhibitor negative control (miScript Inhibitor Negative Control, #1027271, Qiagen) were employed for the transfection. The transfection of inhibitors and inhibitor negative control were accomplished using the HiPerFect Transfection Reagent (#301705, Qiagen) according to the manufacturer’s protocol. After transfection, cells were cultured for 48 h in fresh medium and then exosomes were obtained from AFSC-derived CM. After transfection, expression levels of mature miR-146a and miR-10a in AFSCs and their exosomes were analyzed by qRT-PCR according to the manufacturer’s protocol. Subsequently, the manipulated exosomes were labeled with PKH26 and then directly added into culture medium of CTx-damaged GCs at a concentration of 30 μg ml^−1^ of exosomes proteins.

### Liposome-mediated delivery of miRNAs into CTx-damaged GCs

N-[1-(2,3-Dioleoyloxy)propyl]-N,N,N-trimethylammonium methyl-sulfate (DOTAP) liposomal transfection reagent (#11202375001, Roche) was used for the delivery of microRNAs. MicroRNA mimics (#MSY0000158 for miR-146a and #MSY0000648 for miR-10a, Qiagen) and negative control siRNA (AllStars Negative Control siRNA, #SI03650318, Qiagen) were employed for the study. Liposome/Mimics complexes were prepared according to the manufacturer’s protocol. After the formation of complexes, the liposomes were labeled with PKH26 according to the manufacturers’ instructions and then were directly added into culture medium of CTx-damaged GCs at a final concentration of 0.67 μg nucleic acid with 4 μg DOTAP per milliliter. The weight ratio of DOTAP versus RNAs was 6 to 1.

For *in vivo* ovarian injection, a suspension of 0.2 μg nucleic acid with 1.2 μg labeled liposomes in 5 μl of RNase-free water was injected through a glass pipette (100 μm tip) into each ovary of mice anesthetized with inhaled isoflurane. The weight ratio of DOTAP versus RNAs was 6 to1. CTx-mice with the injection of RNase-free water only, liposome with negative control mixture, liposome with miR-146a mimic, liposome with miR-10a mimic or liposome with both miR146a and miR-10a mimics were designated to CTx-water-, CTx-Lipo-NC-, CTx-Lipo-OE146a-, CTx-Lipo-OE10a- and CTx-Lipo-OE146a and 10a -mice.

### Statistical Analysis

At least three biological replicates were performed for every experiment in this study and all values were represented as the mean ± standard error of the mean (s.e.m.). An unpaired *t* test and ANOVA with Tukey’s multiple comparisons test were used to analyze the data among groups. *P* < 0.05 was defined as a statistically significant difference.

## Additional Information

**How to cite this article**: Xiao, G.-Y. *et al.* Exosomal miR-10a derived from amniotic fluid stem cells preserves ovarian follicles after chemotherapy. *Sci. Rep.* **6**, 23120; doi: 10.1038/srep23120 (2016).

## Supplementary Material

Supplementary Information
