# Supplementary material for: Exosomal miR-10a derived from amniotic fluid stem cells preserves ovarian follicles after chemotherapy
Source: Sci Rep. 2016 Mar 16;6:23120. doi: 10.1038/srep23120 (PMC4793229; doi:10.1038/srep23120)
Supplement: Supplementary Information [file srep23120-s1.pdf]

Supplementary Information

Exosomal miR-10a derived from amniotic fluid stem cells preserves ovarian follicles after chemotherapy

Guan-Yu Xiao, Chun-Chun Cheng, Yih-Shien Chiang, Winston Teng-Kuei Cheng, I-Hsuan Liu, Shinn-Chih Wu

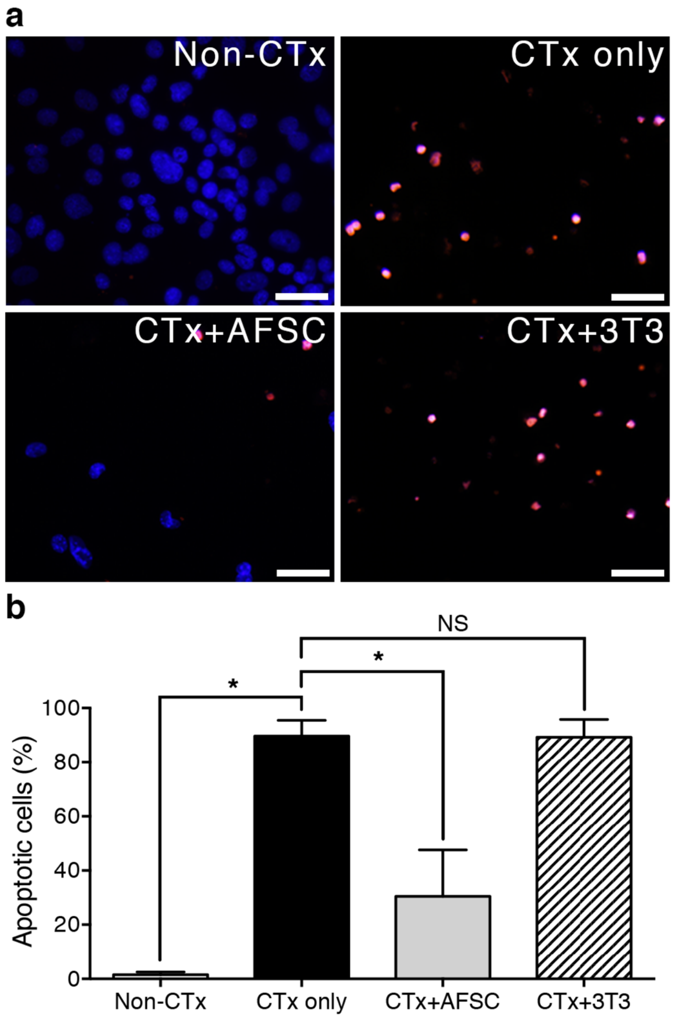

Supplementary Figure 1. AFSCs reduce NM-damaged GCs apoptosis. (**a**) The TUNEL staining images of following groups, GCs cultured without any treatment for 72 hours (Non-CTx). GCs with NM treatment for 24 hours, then removing NM and cultured for another 48 hours (CTx only). After NM treatment for 24 hours, GCs were further transwell co-cultured either with AFSCs (CTx+AFSC) or with NIH-3T3 (CTx+3T3) for another 48 hours. Nucleus was stained by DAPI (blue) while TUNEL positive cells showed as red. Scale bars, 25 μm. (**b**) The apoptotic cells were counted by TUNEL-positive cells in each group. The NM-induced GCs apoptosis was significantly reduced in the presence of AFSCs, compared to other groups. Error bars represent s.e.m. n=3. *$P < 0.05$, NS, not significant; unpaired t test.

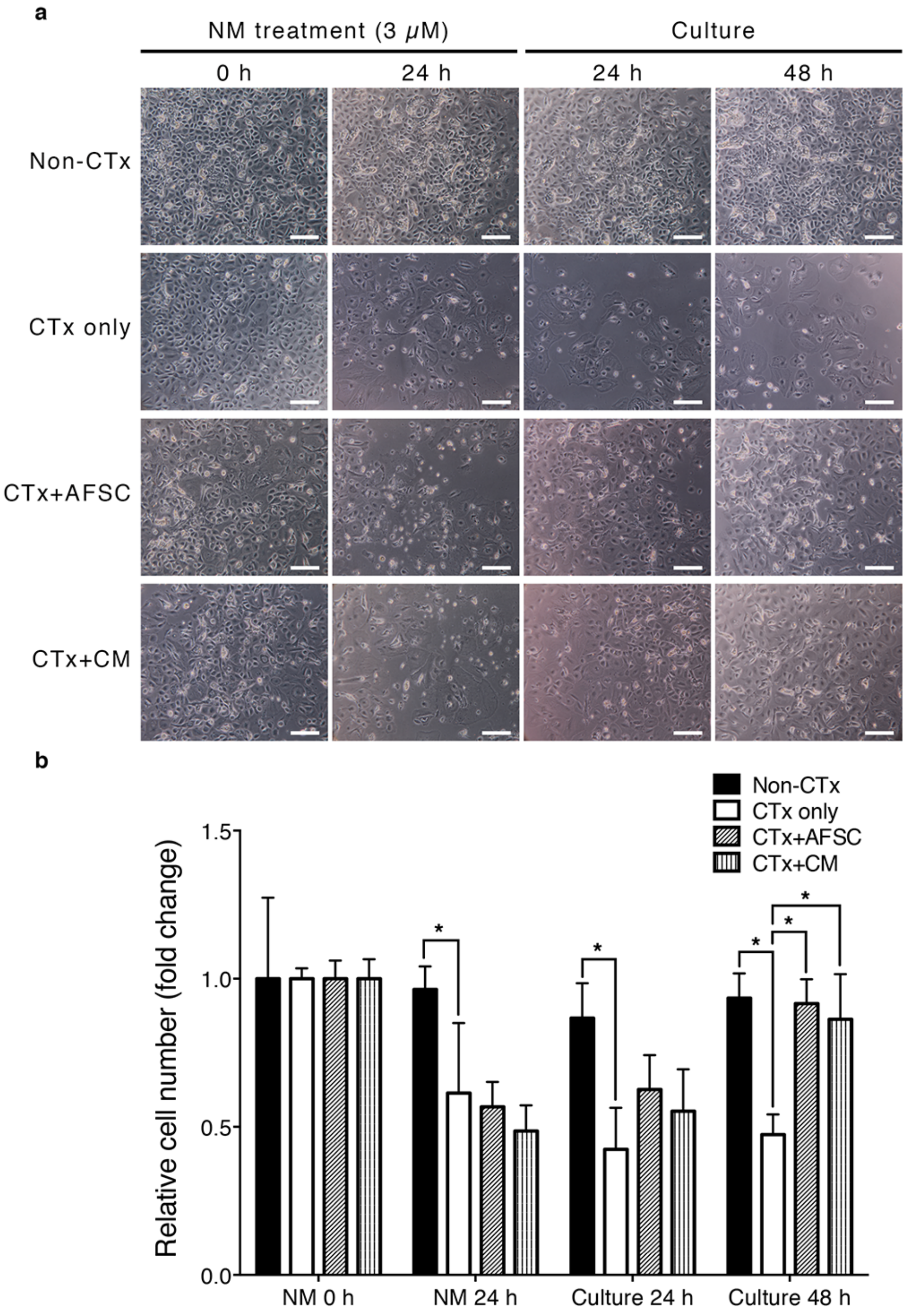

Supplementary Figure 2. AFSCs sustain the viability of NM-damaged GCs via the secretory effect. (**a**) The phase-contrast images of GCs that were followed NM treatment for 24 hours, then removing of NM, and without co-cultured (CTx only) or co-culture with either AFSCs (CTx-AFSC) or CM from AFSCs (CTx-CM) for 24 and 48 hours, except for Non-CTx group. Non-CTx group indicates GCs without NM treatment and without performing co-culture. Scale bars, 100 μm. (**b**) The relative cell number (fold change) in each group at different time points. Error bars represent s.e.m. n=3. *$P < 0.05$; unpaired t test.

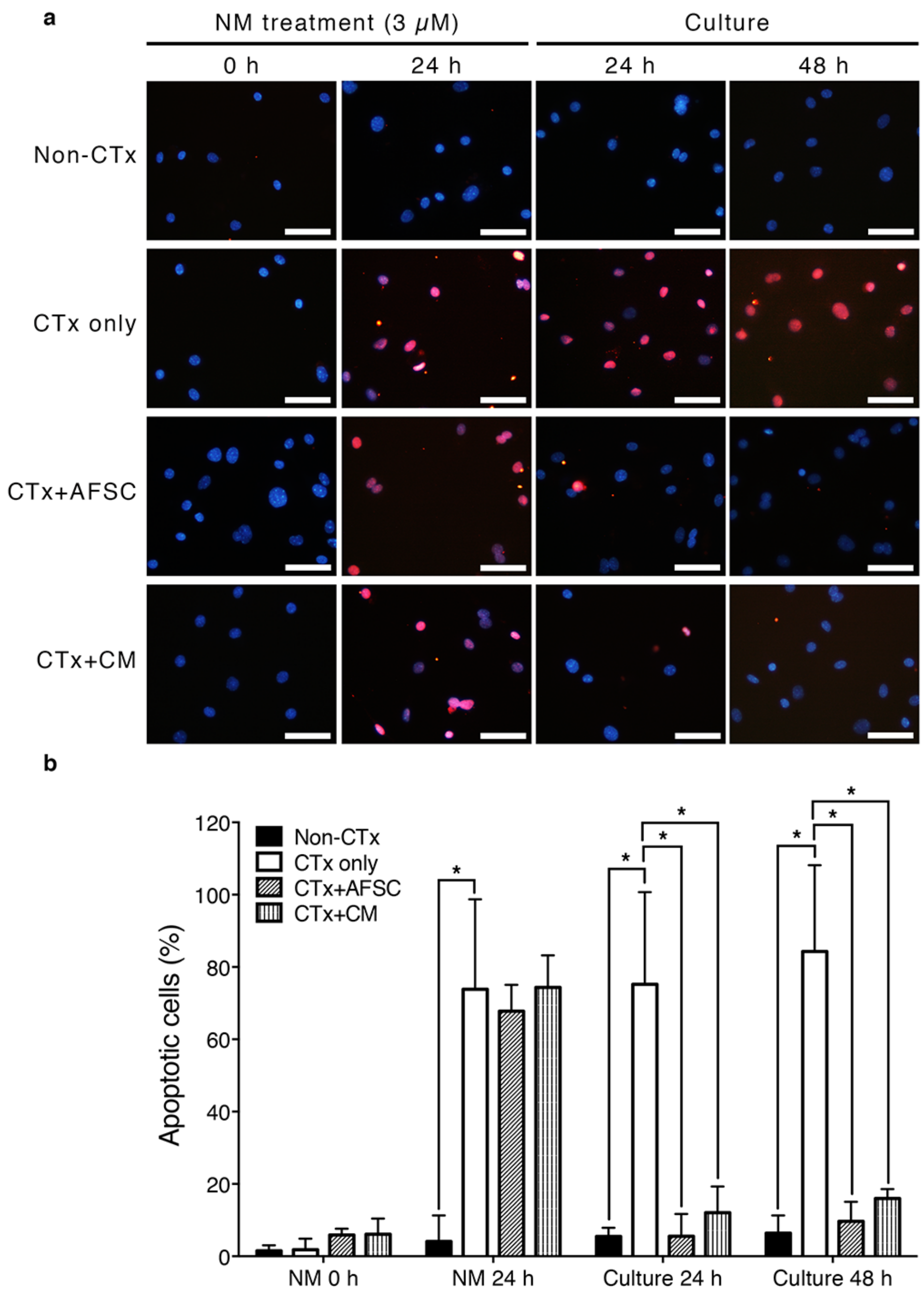

Supplementary Figure 3. AFSCs protect against apoptosis in NM-damaged GCs via the secretory effect. (**a**) The image of TUNEL assay of GCs that were followed NM treatment for 24 hours, then removing of NM, and without co-cultured (CTx only) or co-cultured with either AFSCs (CTx-AFSC) or CM from AFSCs (CTx-CM) for 24 and 48 hours, except for Non-CTx group. Non-CTx group indicates GCs without NM treatment and without performing co-culture. Nucleus was stained by DAPI (blue). TUNEL positive cells (red) indicated the cell undergoing apoptosis. Scale bars, 30 μm. (**b**) The percentage of apoptotic cells in each group at different time points. Error bars represent s.e.m. n=3. *$P < 0.05$; unpaired t test.

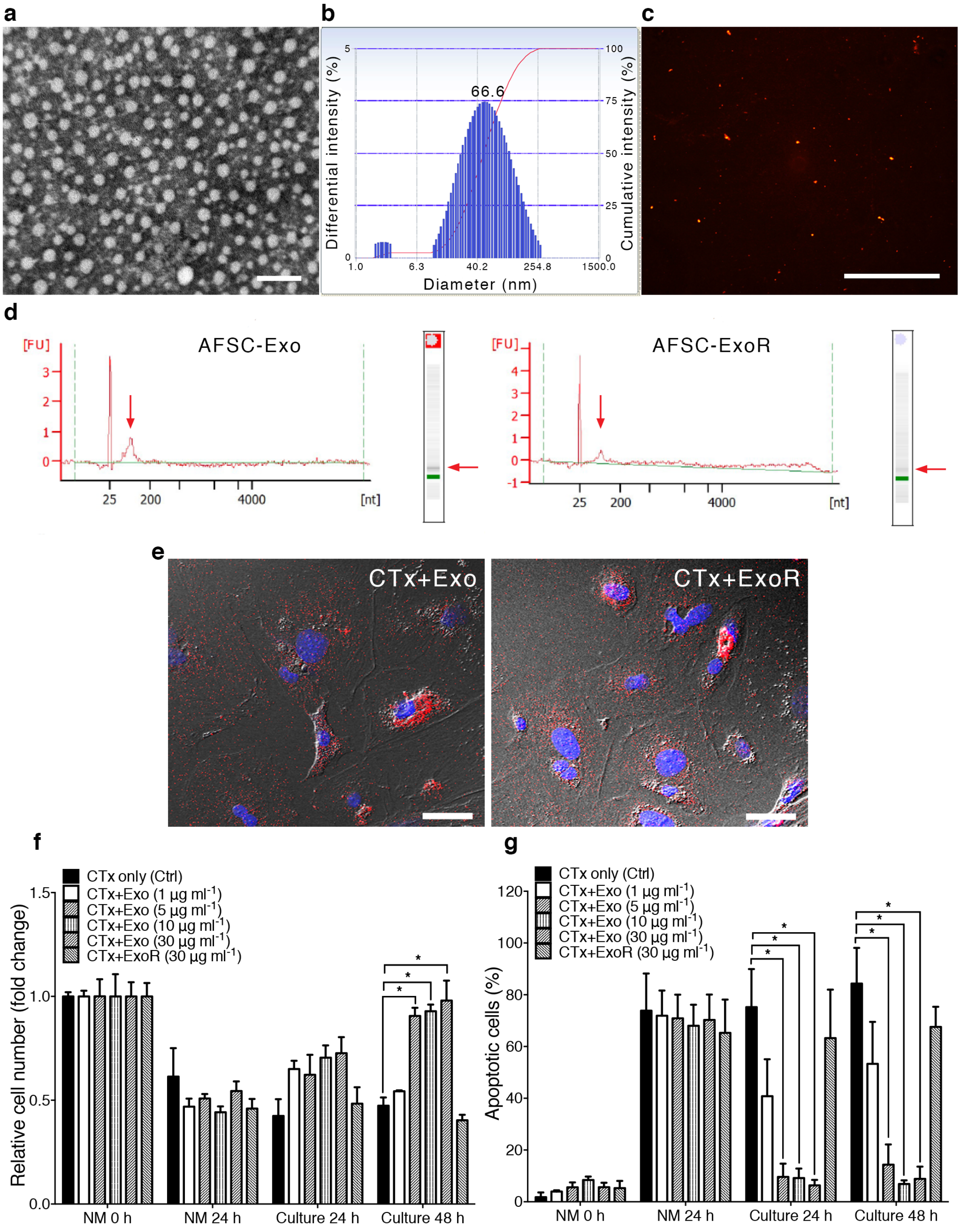

Supplementary Figure 4. Anti-apoptotic effects of AFSC-derived exosomes. (**a**) The representative transmission electron micrograph of AFSC-derived exosomes. Scale bar, 100 nm. (**b**) The size distribution of AFSC-derived exosomes were determined using Nano C Analyzer. (**c**) The representative image of PKH26-labeled AFSC-derived exosomes (red) obtained by fluorescence microscopy. Scale bar, 50 μm. (**d**) The analysis of RNA from AFSC-derived exosomes (AFSC-Exo) and

AFSC-derived exosomes pre-treated with RNase (AFSC-ExoR) by Bioanalyzer. Arrows indicate the RNA content. (**e**) After 3 hours of culture, AFSC-Exo and AFSC-ExoR (both in 30 μg ml^{-1} of exosomes proteins) were incorporated by damaged GCs and distributed in the cytoplasm (red). Nucleus was stained by DAPI (blue). Scale bars, 40 μm. (**f**) The relative cell number (fold change) of damaged GCs cultured with various doses of AFSC-Exo or AFSC-ExoR compared to the CTx only (Ctrl). Error bars represent s.e.m. n=3. *$P < 0.05$; unpaired t test. (**g**) The percentage of apoptotic cells of damaged GCs cultured with various doses of AFSC-Exo or AFSC-ExoR compared to the CTx only (Ctrl). Error bars represent s.e.m. n=3. *$P < 0.05$; unpaired t test.

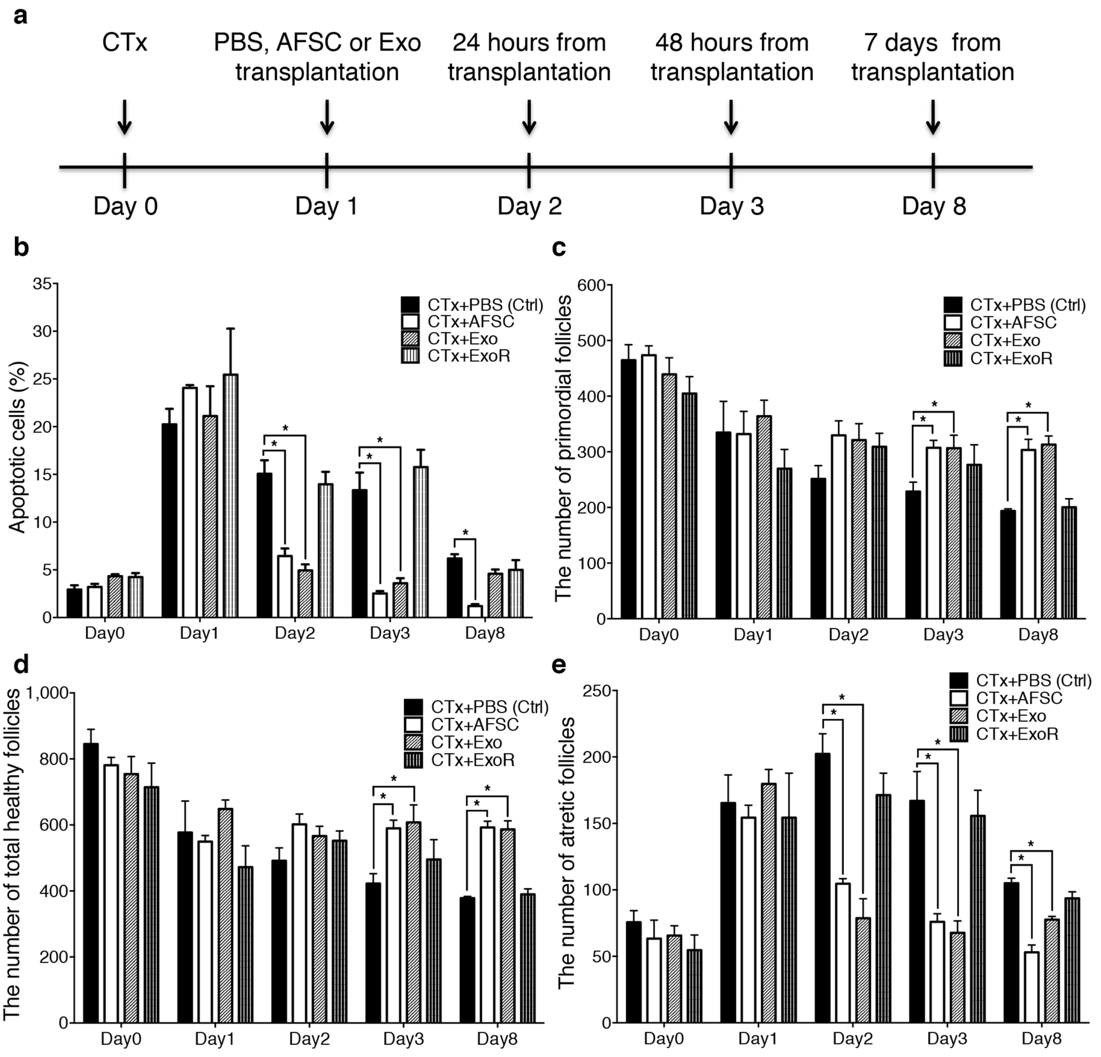

Supplementary Figure 5. AFSC-derived exosomes exert therapeutic effects on ovaries in CTx-mice. (**a**) The schematic diagram of the protocol of CTx administration and followed by treatment with PBS, AFSCs or AFSC-derived exosomes (Exo). (**b**) The percentage of apoptotic cells in ovaries of each group at different time points compared to the CTx+PBS (Ctrl). Exo, AFSC-derived exosomes; ExoR, AFSC-derived exosomes with RNase treatment. Error bars represent s.e.m. n=3. *$P < 0.05$; unpaired t test. (**c-e**) The number of (**c**) primordial, (**d**) total healthy, and (**e**) atretic follicles in ovaries of each group at different time points compared to the CTx+PBS (Ctrl). Exo, AFSC-derived exosomes; ExoR, AFSC-derived exosomes with RNase treatment. Error bars represent s.e.m. n=3. *$P <$ 0.05; unpaired t test.

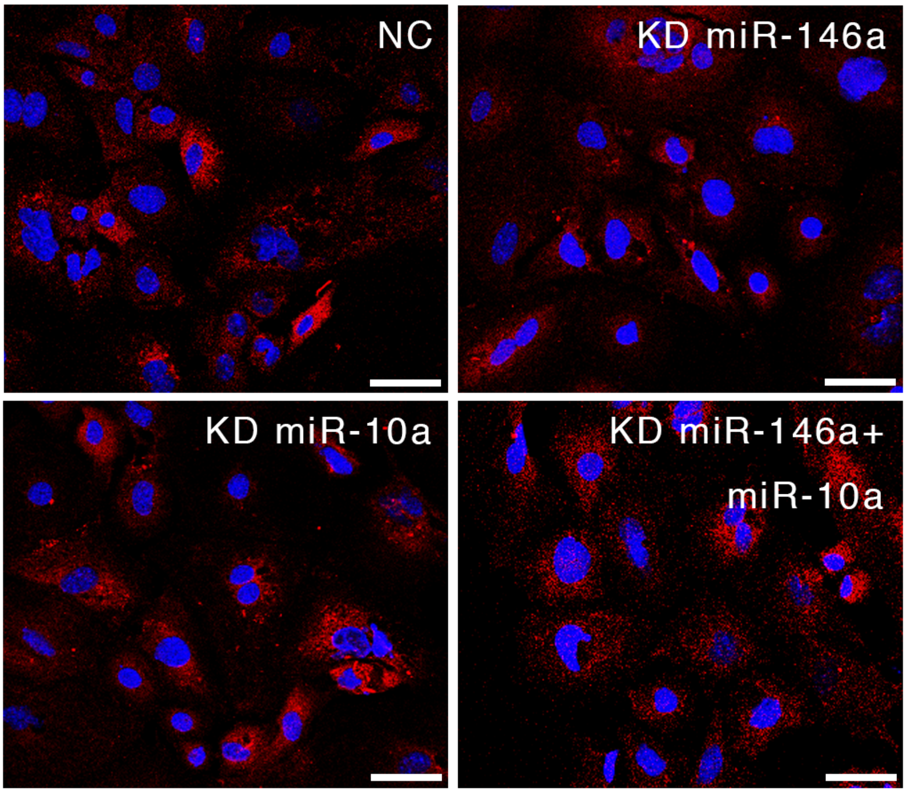

Supplementary Figure 6. Exosomes were incorporated into damaged GCs. After 3 hours of culture, damaged GCs incorporated exosomes derived from AFSCs transfected with inhibitor negative control (NC), miR-146a inhibitor (KD miR-146a), miR-10a inhibitors (KD miR-10a) or both inhibitors (KD miR-146a+miR-10a) into the cytoplasm. Exosomes were labeled by PKH26 (red). Nucleus was stained by DAPI (blue). Scale bars, 40 μm.

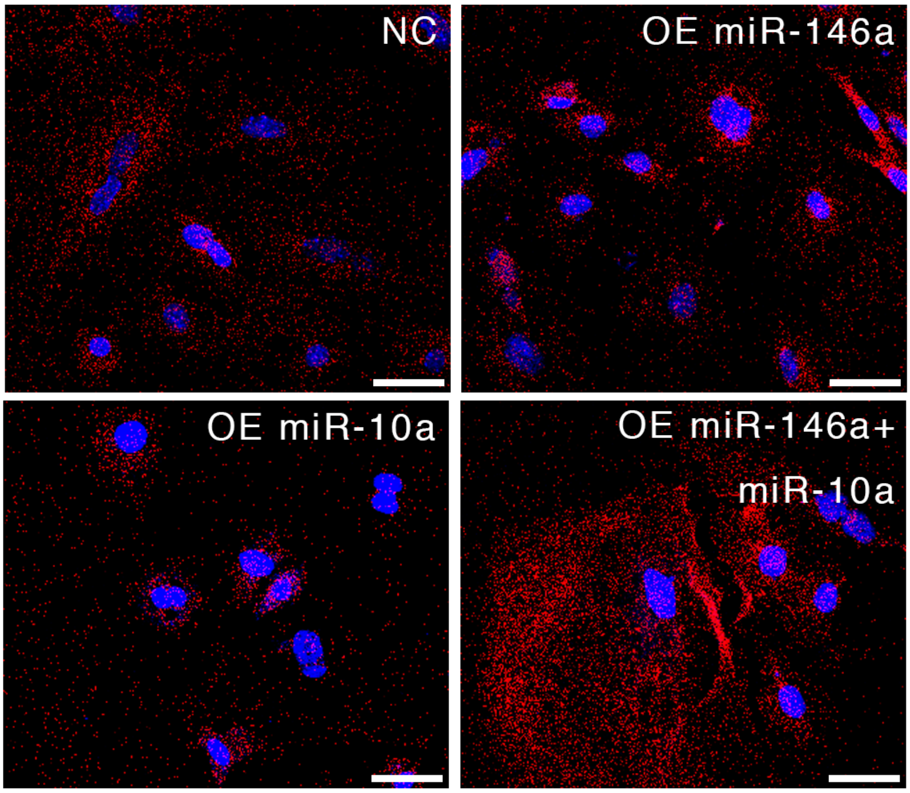

Supplementary Figure 7. Liposomes were incorporated into damaged GCs. After 3 hours of the culture, damaged GCs incorporated liposomes carrying negative control siRNAs (NC), miR-146a (OE miR-146a), miR-10a (OE miR-10a) or both miRNAs (OE miR-146a+miR-10a) into the cytoplasm. Liposomes were labeled by PKH26 (red). Nucleus was stained by DAPI (blue). Scale bars, 40 μm.

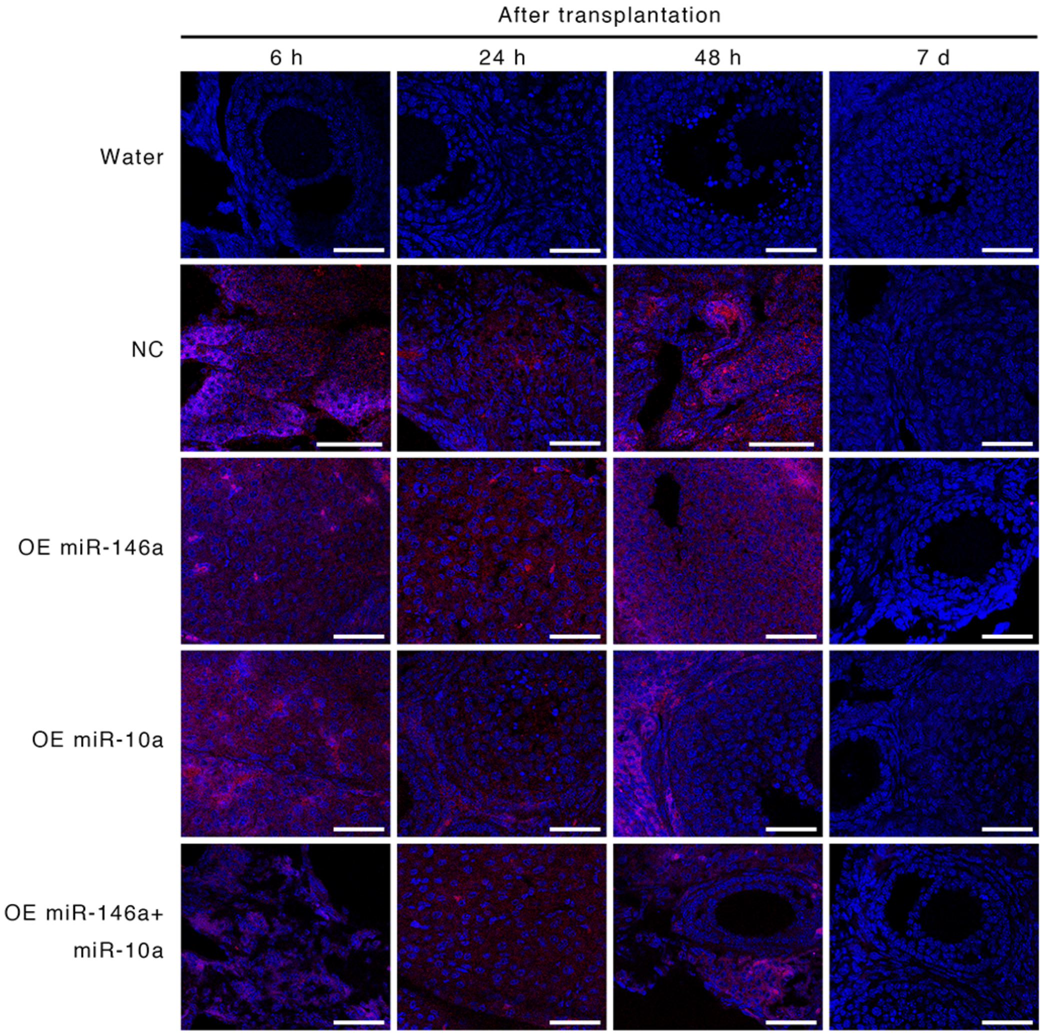

Supplementary Figure 8. Liposomes were incorporated into ovarian cells in CTx-mice. Representative images of ovarian sections of CTx-mice with only water injection, with the injection of liposomes carrying negative control siRNAs (NC), miR-146a (OE miR-146a), miR-10a (OE miR-10a) or both miRNAs (OE miR-146a+miR-10a) at different time points. Liposomes were labeled by PKH26 (red). Nucleus was stained by DAPI (blue). Scale bars, 50 μm.

Supplementary Table 1. A list of miRNAs increased in AFSC-derived exosomes. The miRNA expression level of AFSC-derived exosomes was compared with that of NIH 3T3-derived exosomes. The asterisk (*) indicates that the miRNA relates to anti-apoptosis.

miRNAs	Fold change	miRNAs	Fold change
*mmu-miR-146a-5p	287.20	mmu-miR-181b-2-5p	6.53
*mmu-miR-10a-5p	54.96	mmu-miR-532-5p	6.28
mmu-miR-148a-3p	40.00	mmu-miR-191-5p	6.06
mmu-miR-145-5p	20.70	mmu-miR-540-5p	5.29
mmu-miR-434-5p	18.55	mmu-miR-142-5p	5.26
mmu-miR-138-1-3p	13.50	mmu-miR-666-5p	5.09
mmu-miR-3470b	13.00	mmu-miR-152-5p	5.00
mmu-miR-674-5p	12.00	mmu-miR-16-1-3p	5.00
mmu-miR-374-5p	11.00	mmu-miR-378d	4.962
mmu-miR-205-5p	10.00	mmu-miR-16-2	4.94
mmu-miR-154-5p	9.83	mmu-miR-30d	4.734
mmu-miR-136-5p	9.00	mmu-miR-485	4.6
mmu-miR-138-2-3p	9.00	mmu-miR-15a	4.488
mmu-miR-194-5p	9.00	*mmu-miR-21a	4.484
mmu-miR-382-5p	8.69	mmu-miR-134	4.422
mmu-miR-129-1-3p	8.50	mmu-miR-181a-2	4.412
mmu-miR-194-5p	8.00	mmu-miR-1198	4.333
mmu-miR-339-5p	8.00	mmu-miR-181a-1	4.329
mmu-miR-376a-5p	8.00	mmu-miR-1943	4.2
mmu-miR-5114	8.00	mmu-miR-369	4.2
mmu-miR-150-5p	7.39	mmu-miR-122	4
mmu-miR-224-5p	7.00	mmu-miR-1306	4
mmu-miR-296-5p	7.00	mmu-miR-1843a	4
mmu-miR-99a-5p	7.00	mmu-miR-221	4
mmu-miR-431-5p	6.88	mmu-miR-222	4
*mmu-miR-181b-1-5p	6.64	mmu-miR-3473d	4
mmu-miR-486-3p	6.60	mmu-miR-125b-1	3.962
mmu-miR-486-5p	6.57	mmu-miR-100	3.946

miRNAs	Fold change	miRNAs	Fold change
mmu-miR-340	3.923	mmu-miR-27a	2.5
mmu-miR-3535	3.857	mmu-miR-3057	2.5
mmu-miR-484	3.83	mmu-miR-34a	2.5
mmu-miR-425	3.812	mmu-miR-671	2.5
mmu-miR-126a	3.693	mmu-miR-22	2.469
mmu-miR-127	3.667	mmu-miR-32	2.4
mmu-miR-331	3.667	mmu-miR-30e	2.341
mmu-miR-30a	3.618	mmu-miR-26b	2.339
mmu-miR-144	3.571	mmu-miR-10b	2.274
mmu-miR-215	3.5	mmu-miR-28a	2.25
mmu-miR-125b-2	3.333	mmu-miR-20a	2.231
*mmu-miR-125a	3.143	mmu-miR-214	2.167
mmu-miR-192	3.051	mmu-miR-188	2.143
*mmu-miR-17	3	mmu-miR-26a-2	2.032
mmu-miR-204	3	mmu-miR-26a-1	2.023
*mmu-miR-29b-1	3	mmu-miR-411	2.007
mmu-miR-450a-2	3	mmu-miR-193b	2
mmu-miR-542	3	mmu-miR-1955	2
mmu-miR-673	3	mmu-miR-219a-1	2
mmu-miR-335	2.941	mmu-miR-30b	2
mmu-miR-99b	2.75	mmu-miR-324	2
mmu-miR-24-2	2.737	mmu-miR-345	2
mmu-miR-337	2.667	mmu-miR-3470a	2
mmu-miR-186	2.653	mmu-miR-376b	2
mmu-miR-155	2.6	mmu-miR-450a-1	2
mmu-miR-423	2.589	mmu-miR-8095	2
mmu-miR-877	2.588	mmu-miR-8097	2
mmu-miR-151	2.579		
mmu-miR-181c	2.545		
mmu-miR-93	2.526		

Supplementary Table 2. A list of the primer sequences for qRT-PCR.

Target genes (murine)	Forward Primer (5'-3')	Reverse Primer (5'-3')
Irak1	CAGAGGTGGAACAGCTATCAAG	CATTGGGCAAGAAGCCATAAAC
Traf6	AGCTGTCCTCTGGCAAATATC	GTTGGGCAGTCCAGATCATAA
Bcl2l11 (*Bim)*	GAGCTGGGAGTCTTGTGTACTA	GCCCATATGCTGGGTGTATTT
Casp9	CGACCTGACTGCCAAGAAA	GAGAGGATGACCACCACAAAG
Gapdh	CATGGCCTTCCGTGTTCCT	GCGGCACGTCAGATCCA
